# Critical Period Regulation by Thyroid Hormones: Potential Mechanisms and Sex-Specific Aspects

**DOI:** 10.3389/fnmol.2019.00077

**Published:** 2019-04-05

**Authors:** Gervasio Batista, Takao K. Hensch

**Affiliations:** ^1^Center for Brain Science, Department of Molecular Cellular Biology, Harvard University, Cambridge, MA, United States; ^2^FM Kirby Neurobiology Center, Department of Neurology, Boston Children’s Hospital, Boston, MA, United States; ^3^International Research Center for Neurointelligence, University of Tokyo Institutes for Advanced Study, Tokyo, Japan

**Keywords:** parvalbumin cell, perineuronal nets, oxidative stress, Otx2, acetylcholine

## Abstract

Adequate perinatal levels of thyroid hormones (THs) are required for normal brain function and development. Studies in non-mammalian species suggest that TH might be involved in the regulation of critical periods (CPs) of heightened plasticity. Yet, it is largely unknown what mechanisms controlling such CPs might be under TH regulation. Here, we briefly review the influence of TH in early life across evolution. We discuss possible links between TH and known circuit and/or molecular mechanisms determining the timing of CPs of heightened brain plasticity. We focus on the role of parvalbumin-positive (PV) interneurons since their maturation defines CP onset and closure. Specifically, abnormal PV circuits are associated with low perinatal levels of TH, possibly because thyroid hypofunction may increase oxidative stress and/or dysregulate Otx2-mediated maturation of neuroprotective perineuronal nets. In addition, the level of cholinergic transmission is important for CP plasticity. Potentially, TH levels could affect gain changes in cholinergic transmission that can alter brain development. We believe that understanding how TH impacts CPs of circuit refinement will shed light onto the principles underlying normal developmental trajectories. Given that the thyroid gland expresses estrogen and androgen receptors, its activity can potentially be regulated differently between the sexes, contributing to sexually dimorphic behaviors.

## Introduction

Thyroid hormones (THs) adjust a myriad of metabolic variables to suit environmental demands (Mullur et al., 2014). In the brain, TH milieu is particularly important for appropriate pre- and postnatal development and regulation of crucial cellular events (Zoeller, 2010). Thyroid gland hypofunction in pregnant women, for example, significantly increases autism risk (Román et al., 2013) and low perinatal TH levels are associated with persistent cognitive impairments and attentional deficits (reviewed in Salerno et al., 2016). Thus, it is clear that Thyroxine (T4) production, its conversion to Triiodothyronine (T3) and activation of Thyroid hormone receptors (THr) are vital processes to guarantee normal brain maturation. These events can be spatiotemporally regulated across development to control gene expression and shape brain organization. Precise orchestration of TH-signaling during windows of heightened plasticity might be particularly important. These critical periods (CPs) potently sculpt brain function in response to early experience (Hensch, 2004) and may be dysregulated in psychiatric conditions (Marín, 2016). Hence, understanding the impact of TH on CP timing might yield new insights into the biological basis of brain disorders.

Here, we first review CPs where TH is known to play a major role. Second, we explore multiple molecular pathways by which CP plasticity could be linked to TH actions. Third, we consider regulation of thyroid gland function by sex hormones. Finally, we point to future directions that could shed light on the role of TH in the juvenile brain.

## Achieving Spatiotemporal Specificity Through Local Regulation of TH-Signaling

**Figure 1:**
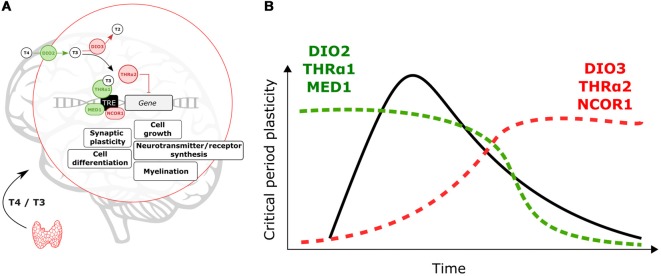
Molecular events controlling locally thyroid hormone (TH)-signaling. **(A)** Deiodinases, THrα-variants and transcription co-regulators can enhance (green)/repress (red) T3-dependent transcription. **(B)** Expression levels of DIO2, THRα1 and MED1 are predicted to increase to support critical period (CP) onset and plasticity. After the CP breaks of TH-action (DIO3, THRα2 and nuclear receptor coregulator 1, NCOR1) could contribute to decreasing TH-signaling and T3-related changes in gene expression.

Changes in local T3 synthesis in the hypothalamus of birds mediate photoperiodic responses (Yoshimura et al., 2003). It is thus possible that on a developmental time scale local TH-signaling is achieved across cortical areas. As shown in Figure 1A, TH signaling is mediated by T3 the transcriptionally active hormone when bound to TH nuclear receptors (THr; Mullur et al., 2014). In the developing brain, the type-2 deiodinase (DIO2) locally converts T4 into T3 (Bárez-López et al., 2018). Instead, type-3 deiodinase (DIO3) is in charge of reducing cellular levels of T3 (Gereben et al., 2008). Levels of DIO2 and DIO3 can finely tune T3 availability at specific times during brain development (Figure 1B).

There are two types of THr, THrα and THrß. The THrα is widely expressed across the brain while THrß is predominantly expressed in subcortical brain areas (Flamant et al., 2017). Here, we will focus on THrα but it is possible that combinatorial effects of both receptor types shape brain maturation. Alternative splicing gives rise to two THrα variants, α1 and α2 (Hodin et al., 1990). T3-dependent transcription is mediated by THrα1 (Hodin et al., 1990). In contrast, the THrα2 variant does not bind to T3 and represses T3-dependent transcription (Hodin et al., 1990; Mullur et al., 2014). TH-signaling during CPs can be enhanced, or reduced, by altering expression levels of THrα1 and THrα2 (Figure 1).

In addition, transcription coregulators (enhancers/repressors) can tune T3-dependent transcription. The nuclear receptor coregulator 1 (NCOR1) is particularly important to regulate TH actions *in vivo* (Shimizu et al., 2015). Yet the expression of this repressor during brain development is poorly understood. The coactivator MED1 (Mediator of RNA polymerase II transcription subunit 1) enhances T3-dependent transcription (Park et al., 2005), which could contribute to enhance TH effects and oppose NCOR1 actions (Figure 1).

As shown in Figure 1, we expect that local enhancement of TH signaling is achieved early and during CPs through upregulation of DIO2, THRα1 and MED1. On the other hand, DIO3, THRα2 and NCOR1 upregulation upon CP closure can act as brakes for TH actions and associated changes in gene expression. We propose that from regulation of these elements, developmental changes in TH-signaling can contribute to the timing and plasticity of CPs. Moreover, it is possible that genetic and environmental insults disrupt local changes in TH signaling in early life to have major impacts on maturational brain trajectories.

## Time-Constrained Effects of Thyroid Hormones During Development

### TH-Signaling During CPs in Non-brain Tissue

Brief periods of high TH sensitivity characterize the maturation of body organs. A transient increase (between P5 and 13) in the THrα1/THrα2 ratio determines a CP for TH-dependent expression of hexose transporters within the jejunum of rats (Mochizuki et al., 2007). Similarly, renal development is also under TH control during a short period of time (Tan et al., 1997). Before the kidney is innervated, THr regulates the expression of the adrenoreceptor α1, which transduce neurotrophic signals, exclusively within the first 3 weeks postpartum (Tan et al., 1997).

### Time-Constrained Effects of TH in the Retina

In zebrafish, the thickness of the inner retinal layers responds to TH blockade within 65–66 h post fertilization but not in fully mature subjects (Reider and Connaughton, 2014). This CP was shown to be sensitive to temperature shifts (Reider and Connaughton, 2014), but the mechanisms underlying temperature sensitivity of TH actions remain unknown. Notably, the differential expression of THr-specific retinal layers is reported in chickens (Sjöberg et al., 1992) with limited explanation.

### TH Actions and Cerebellar Development

Normal levels of perinatal TH are required for the correct development of cerebellar circuitry in rodents (Koibuchi, 2008). The dendritic complexity of Purkinje cells is heavily compromised in rats with low perinatal TH (Nicholson and Altman, 1972). Cerebellar connections might also be under TH control during the CP given that specific subsets of THr are expressed in different cells (Koibuchi, 2008). These findings indicate that dysregulation of thyroid function early in life may have a large impact on cerebellum-mediated motor function. Aberrant cerebellar development could lead to further cognitive impairments where this large processing structure is suspected to participate. The emergence of anomalous cerebellar-cortical communication during CPs has been linked to autistic disorders (Wang et al., 2014). Notably, dysregulation of TH signaling is also found in patients with autism (Yuen et al., 2015).

## Early Thyroid Hormone Levels Regulate Complex Behaviors

Thyroid function around birth is important for the development of a variety of behaviors across vertebrates. Perinatal TH are fundamental in humans (Törel Ergür et al., 2012), rodents (van Wijk et al., 2008), birds (Yamaguchi et al., 2012) and fish (Lema and Nevitt, 2004) to support different behaviors. Comparing the role of TH across these species could contribute to better understand their developmental role.

### Perinatal Thyroid Hypofunction Has Long-Lasting Effects

Cognitive, verbal and motor deficits characterize patients with congenital hypothyroidism (Oerbeck et al., 2003; Kempers et al., 2006). Moreover, subclinical hypothyroidism of children and adolescents correlates with attention deficits (Törel Ergür et al., 2012). In rodents, low levels of TH lead to altered locomotion (Sadamatsu et al., 2006; van Wijk et al., 2008), impaired spatial memory (Sadamatsu et al., 2006) and reduced anxiety (Darbra et al., 1995). Genetic ablation of THrα and ß modify anxiety- and fear-related behaviors (Guadaño-Ferraz et al., 2003; Vasudevan et al., 2013). Interestingly, neonatal induced hypothyroidism also increases the number audiogenic seizures in adulthood (Yasuda et al., 2000), suggesting that TH deficits early in life may trigger a long-term imbalance between excitatory and inhibitory transmission.

### Thyroid Hormone Functions During CPs in Non-mammalian Species

The role of TH during CPs is evolutionary conserved. Imprinting, the formation of a long-term memory within a sensitive period (Horn et al., 2001; Jin et al., 2016), requires activation of THr as demonstrated in chickens (Yamaguchi et al., 2012). This form of learning only occurs within the first 3 days after hatching unless chickens are treated with TH and the CP is reopened (Yamaguchi et al., 2012).

In salmon, where olfactory imprinting occurs (Bett et al., 2016), TH peak at the onset of the CP for memory formation (Lema and Nevitt, 2004). This has not been directly linked to experience-dependent behavioral plasticity, but T4 and T3 control cellular proliferation within the olfactory epithelium during the sensitive period (Lema and Nevitt, 2004). In turn, reorganization of peripheral olfactory processing could support imprinting by facilitating detection of specific odors.

Rising levels of TH have also been reported during the CP for song learning (Yamaguchi et al., 2017). While this correlation has not been shown to be causal, it is interesting that high levels of TH are present at the onset of several CPs (Lema and Nevitt, 2004; Yamaguchi et al., 2012, 2017). Thus, taken together the evidence gathered from mammalian and non-mammalian species supports that TH is highly active within the time-constrained developmental windows and regulate the organization of diverse and complex behaviors.

## CP Regulation Through Thyroid Hormones: Potential Links

When vision from one eye is deprived during a specific window of time, binocular neurons in primary visual cortex (V1) lose their responsiveness to that visual input (Hubel and Wiesel, 1970). This process can lead to long-term blunted visual acuity, known as amblyopia (McKee et al., 2003). The study of ocular dominance plasticity (ODP) has shed light onto several circuits and molecular mechanisms controlling CPs. Here, we try to establish possible connections between such mechanisms and THr activation.

### TH-Dependent Maturation of GABAergic Transmission Circuits

Pharmacological and genetic manipulation of inhibition bidirectionally affects CP timing for ODP (Hensch et al., 1998; Iwai et al., 2003; Hensch, 2005; Chattopadhyaya et al., 2007). While benzodiazepines can open V1 CP prematurely (Hensch et al., 1998), genetically-induced reduction of GABA synthesis delays ODP (Iwai et al., 2003; Chattopadhyaya et al., 2007). Several studies support a link between GABAergic transmission modulation and TH (reviewed in Wiens and Trudeau, 2006). Hence, TH could control CP timing through regulation of inhibitory transmission.

Glutamate is converted into GABA by the glutamate acid decarboxylate (GAD) enzyme. In vertebrates, the isoforms GAD65 and GAD67 mediate GABA synthesis (Fenalti et al., 2007). *In vitro* and *in vivo* studies demonstrated that TH regulates GAD expression in the brain (Wiens and Trudeau, 2006). Interestingly, GAD65 and GAD67 expression is more sensitive to TH around birth compared to adulthood (Wiens and Trudeau, 2006) and is spatiotemporally regulated in rats (Popp et al., 2009). Whether the spatiotemporal control of GAD expression and function responds to differential TH-signaling across brain regions remains to be tested.

In addition to GABA synthesis, TH can influence other aspects of inhibitory networks. Early in development TH shapes the morphology and connectivity of GABAergic cells (Westerholz et al., 2013). These effects are mediated by trkb and mTOR pathways (Westerholz et al., 2013). Interestingly, mTOR is also recruited by TH to promote behavioral plasticity during the CP for imprinting in chickens (Batista et al., 2018). Thus, TH/mTOR signaling appears to play a major role during early life, potentially, through regulation of GABAergic transmission. Supporting this view, a recent study showed that TH control a developmental switch in GABAa/GABAb receptors to open the CP for imprinting in chicks (Aoki et al., 2018).

### PV Maturation Requires Normal Levels of TH

**Figure 2:**
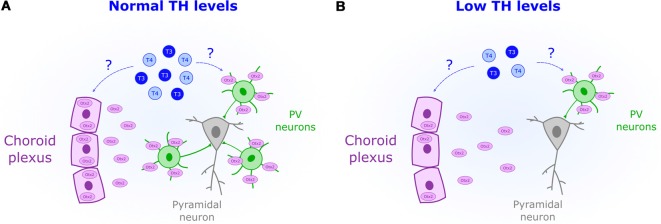
Reduced perinatal levels of THs (blue) are associated with a decreased presence of parvalbumin-positive neurons (PV). **(A)** Normal levels of TH could act as permissive signals for CP onset by increasing Otx2 (purple) synthesis in the choroid plexus. **(B)** Downregulation of Otx2 expression in the choroid plexus could mediate the observed impairment in PV maturation under low levels of TH.

Increased inhibitory transmission mediates CP opening in V1 (Hensch et al., 1998). Yet ODP plasticity specifically involves the activation of GABA-receptors (GABARs) containing the subunit α1 (Fagiolini et al., 2004). This subunit is mostly expressed, and targeted, by parvalbumin-positive interneurons (PV; Fagiolini et al., 2004). The experience-dependent maturation of PV circuits opens the CP in V1 (Sugiyama et al., 2008) and plays a similar role in other brain areas (Spatazza et al., 2013). PV cells are particularly sensitive to perinatal TH levels. Hence, TH impact on PV neuron maturation might be crucial to set CP timing not only in V1 (Figure 2). In rat neocortex, perinatal-induced hypothyroidism decreases PV expression (Berbel et al., 1996; Royland et al., 2008). TH-mediated impairments in PV expression has been also found in the hippocampus of rats, an area extremely responsive to perinatal TH levels (Gould et al., 1990b; Gilbert et al., 2007; Sawano et al., 2013). Other brain regions where TH developmentally regulate PV expression include the hypothalamus (Harder et al., 2018) and the striatum (Bode et al., 2017). Given the importance of PV circuits in CP regulation, it is likely that perinatal TH deficits disrupt CP timing across brain regions, which can be adequately tested in sensory systems.

### Otx2 Expression Is Regulated by TH

The transcription factor Otx2 is crucial for non-cell autonomous PV maturation (Sugiyama et al., 2008). Experience-dependent accumulation of Otx2 derived from the retina (Sugiyama et al., 2008) and choroid plexus (Spatazza et al., 2013) triggers the onset of the CP. Two facts relate TH and Otx2. Transthyretin, the protein transporting TH into the cerebrospinal fluid, is synthesized by the choroid plexus (Richardson et al., 2015). Yet, more importantly, Otx2 production can be regulated by THr activation as shown in midbrain stem cells (Chen et al., 2015). Whether TH controls Otx2 synthesis in the choroid plexus remains unknown. But given its role in midbrain (Chen et al., 2015), it is possible that TH acts as a permissive signal to set the onset of the CP by enhancing Otx2 production and PV maturation (Figure 2).

### Oxidative Stress Is Affected by Thyroid Function

**Figure 3:**
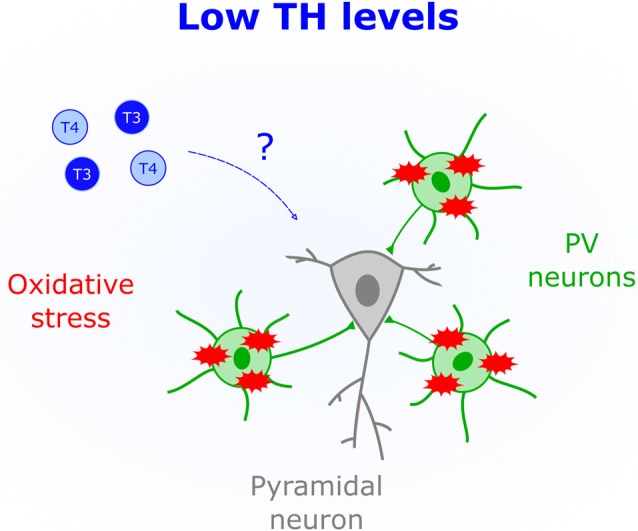
Low levels of THs (blue) correlate with increased oxidative stress. The schematic shows that parvalbumin-positive cells (PV), but not pyramidal cells, are particularly sensitive to oxidative stress, which could mediate brain deficits triggered by low perinatal TH levels.

Fast-spiking properties of PV cells impose a heavy metabolic burden rendering them susceptible to oxidative stress (Cabungcal et al., 2013). This particular feature is thought to underlie their impairment in human patients with mental illness, such as psychosis (Andreazza et al., 2008; Yao and Keshavan, 2011). Unchecked oxidative stress within PV cells also leads to aberrantly extended V1 plasticity (Morishita et al., 2015), thus demonstrating the importance of redox regulation for normal CP timing. Human studies indicate that hypothyroid patients (Resch et al., 2002) and subclinical low levels of TH (Santi et al., 2012) are associated with a surge in oxidative stress. Mechanisms underlying TH-mediated oxidative stress are not fully elucidated; however, it has been suggested that hypothyroidism reduces the presence of antioxidants (Mancini et al., 2016). One such agent, glutathione, impacts CP duration (Morishita et al., 2015). Therefore, TH actions in early life could include regulating antioxidant synthesis within PV circuits (Figure 3).

### TH-Mediated Control of Cholinergic Transmission

**Figure 4:**
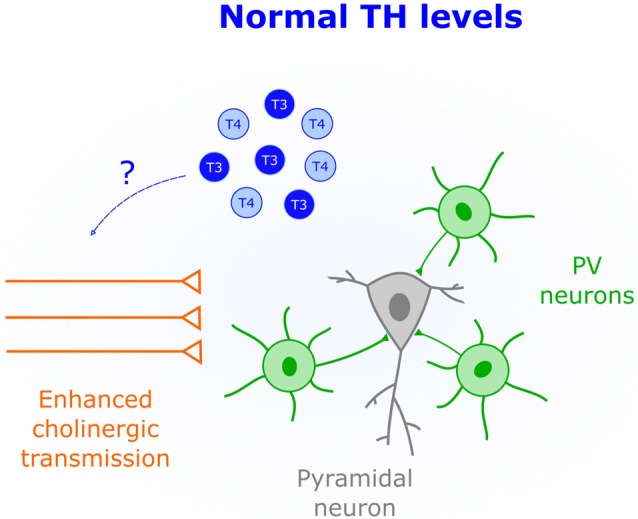
Enhanced cholinergic transmission is characteristic of CPs. An important role of TH (blue) early in life could be to increase the acetylcholine synthesis and release.

Activation of cholinergic and muscarinic receptors by acetylcholine underlies synaptic plasticity and attention (Picciotto et al., 2012). Thus, changing the gain of cholinergic transmission over development appears is a powerful mechanism that constrains plasticity to specific time windows. Indeed, in V1 the protein Lynx1 dampens the activity of cholinergic receptors to close the CP (Morishita et al., 2010). Interestingly, non-pathological low levels of TH correlate with attention deficits in children and adolescents (Törel Ergür et al., 2012). This effect might be due to decreased cholinergic transmission since cell-specific optogenetics demonstrated that cholinergic inputs play an important role in attention (Luchicchi et al., 2014). It is thus possible that early life TH levels influence cholinergic transmission to regulate plasticity. Additional evidence supporting this view comes from *in vitro* studies where both release and synthesis of acetylcholine is enhanced by T4 application (Landa et al., 1991). Additionally, cholinergic deficits in the Snell Dwarf Mouse are corrected by T4 injections (Fuhrmann et al., 1986), suggesting that TH-mediated modulation of cholinergic transmission occurs *in vivo*. Further research is needed to establish a causal link between TH-mediated changes in cholinergic signaling and CP regulation (Figure 4).

## Influences of Sex Hormones on Thyroid Function

Can the developmental role of TH contribute to sex differences in the brain? Is the thyroid gland sensitive to gonadal hormones? Do male and female thyroid glands respond differently to environmental insults? These sorts of questions remain largely unanswered, but they might be critical to uncovering how the role of TH during CPs could relate to sex-differences in the brain.

### Sex-Specific Behavioral Plasticity in Birds During CPs

Filial imprinting in birds is tightly associated with TH levels (Yamaguchi et al., 2012). Historically, imprinting studies in chickens have not distinguished between male and female learning mechanisms. However, subtle differences have been reported (Miura and Matsushima, 2012). For example, male chickens have stronger innate preferences for biologically moving objects compared to females. It remains unclear whether these differences arise from TH-dependent organization of imprinted circuits or not. To address this, it would be important to assess whether thyroid function is comparable across sexes at hatching. In Zebra Finches, where males but not females develop a song over a CP, TH levels rise earlier in males (Yamaguchi et al., 2017). This increase in TH levels correlates with the appearance of PV in regions controlling song production (Balmer et al., 2009). Hence, it is possible that dimorphic TH levels contribute to sex-specific behaviors later controlled by PV maturation.

### Gonadal Steroids Control Thyroid Gland Growth

Androgens and estrogens enhance thyroid stimulating hormone (TSH) effects on the thyroid gland in a sex-specific manner (Banu et al., 2002; Sakhila Banu and Aruldhas, 2002). Receptors for gonadal steroids have been reported to enhance thyroid gland growth and function (Sakhila Banu and Aruldhas, 2002). Thus, when the production of gonadal hormones increases around puberty, thyroid gland growth is heightened (Sakhila Banu and Aruldhas, 2002). In turn, this may generate dimorphic developmental trajectories in males and females since the latter enter adolescence earlier (Brenhouse and Andersen, 2011). Yet it is unknown how androgens and estrogens interact with TH to sculpt the brain and whether this interaction underlies sexual dimorphism.

### Sex-Specific Remodeling of Hippocampal Circuits

In mouse hippocampus, perinatal TH injections trigger sex- and sub-region specific effects (Gould et al., 1990b). Pyramidal cells in the CA3 area of male mice treated with thyroxine around birth, show fewer primary dendrites than females receiving the same treatment (Gould et al., 1990b). Notably, these structural changes could be tracked throughout life and are not detected in the nearby CA1 subregion (Gould et al., 1990b). Therefore, in addition to changes in thyroid function in adolescence, there is already a sexually dimorphic sensitivity to TH levels at birth that can cause long-lasting reorganization of neural circuits.

### Thyroid Dysfunction Prevails in Women

Women exhibit a higher risk for thyroid malfunction compared to men (Bauer et al., 2014). The underlying mechanism for this susceptibility is under investigation. In rats, it has been demonstrated that an estrogen receptor-mediated increase in oxidative stress is responsible for the prevalence of thyroid dysfunction in females (Fortunato et al., 2014). This is particularly important considering that patients with bipolar disorders with comorbidity of thyroid problems is higher in women (Bauer et al., 2014). Further investigation is required to clarify the role of TH in female bipolar disorders (Bauer et al., 2014). One possible scenario is that sex-specific vulnerability to some psychiatric diseases arises from abnormal thyroid gland function in women.

### Environmental Insults Can Trigger Sexually Dimorphic Thyroid Impairments

Neurotoxins affect the thyroid gland differently in men and women, thus leading to TH-mediated impairments in neurocognitive performance. Exposure to the industrial surfactant perfluorooctanoic acid is highly associated with thyroid dysfunction in women (Melzer et al., 2010). Similarly, exposure to radiation correlates with thyroid abnormalities in both men and women but is more prevalent within the latter (Lyon et al., 2006). These studies point to women as having an enhanced susceptibility to environmental risk factors. However, other unknown factors could be linked to thyroid dysfunction in men. For example, breathing second-hand smoke acutely enhances TH production in men (Flouris et al., 2008). Therefore, it might be the case that certain neurotoxins have sex-specific impacts on thyroid physiology indirectly leading to TH-mediated brain deficits.

## Concluding Remarks and Future Directions

Shifts in the onset and closure of windows of enhanced plasticity could lead to characteristic features of some neuropsychiatric disorders such as autism (LeBlanc and Fagiolini, 2011), schizophrenia and bipolar disorder (Yao and Keshavan, 2011; Morishita et al., 2015). A deeper understanding of the interaction between gonadal hormones and thyroid function can shed light onto novel principles underlying sex-differences in brain organization. Future research might fill the gap between CPs regulation and TH signaling. In addition, the circuits that are sensitive to perinatal T4 and T3 levels need to be identified. Given that hypothyroidism has been associated with functional and structural alterations within the cerebellum (Koibuchi, 2008), hippocampus (Gould et al., 1990a; Gilbert et al., 2007), cortex (Royland et al., 2008) and subcortical nuclei (Yasuda et al., 2000), TH are expected to have global impacts on brain functions. Recently, several studies suggest a link between gut microbiota and the brain (Dinan and Cryan, 2017). THs might also be important players in this poorly understood relationship. Research in humans and rodents suggest that intestinal microbiota influences thyroid function, the availability of iodine and selenium and T4 absorption (Virili and Centanni, 2015). This could be relevant not only for adult brain function but also early in life during the CPs of plasticity. In summary, neonatal thyroid function appears to be tightly related to developmental events that are constrained to specific windows of time across evolution. Yet more research is needed to understand how THs might regulate the mechanisms controlling CPs timing and plasticity.

## Author Contributions

GB and TH wrote the manuscript.

## Conflict of Interest Statement

The authors declare that the research was conducted in the absence of any commercial or financial relationships that could be construed as a potential conflict of interest.
